# Eliminating roles for T-bet and IL-2 but revealing superior activation and proliferation as mechanisms underpinning dominance of regulatory T cells in tumors

**DOI:** 10.18632/oncotarget.5584

**Published:** 2015-09-10

**Authors:** Emily J. Colbeck, James P. Hindley, Kathryn Smart, Emma Jones, Anja Bloom, Hayley Bridgeman, Rhoanne C. McPherson, Darryl G. Turner, Kristin Ladell, David A. Price, Richard A. O'Connor, Stephen M. Anderton, Andrew J. Godkin, Awen M. Gallimore

**Affiliations:** ^1^ Institute of Infection Immunity and Biochemistry, Cardiff University School of Medicine, Cardiff, UK; ^2^ MRC Centre for Inflammation Research, Centre for Multiple Sclerosis Research and Centre for Immunity Infection and Evolution, University of Edinburgh, Edinburgh, UK

**Keywords:** Treg, Cancer, proliferation, T-bet, CD69, Immunology Section, Immunity, Immune response

## Abstract

Foxp3^+^ regulatory T cells (Tregs) are often highly enriched within the tumor-infiltrating T cell pool. Using a well-characterised model of carcinogen-induced fibrosarcomas we show that the enriched tumor-infiltrating Treg population comprises largely of CXCR3^+^ T-bet^+^ ‘T_H_1-like’ Tregs which are thymus-derived Helios^+^ cells. Whilst IL-2 maintains homeostatic ratios of Tregs in lymphoid organs, we found that the perturbation in Treg frequencies in tumors is IL-2 independent. Moreover, we show that the T_H_1 phenotype of tumor-infiltrating Tregs is dispensable for their ability to influence tumor progression. We did however find that unlike Tconvs, the majority of intra-tumoral Tregs express the activation markers CD69, CD25, ICOS, CD103 and CTLA4 and are significantly more proliferative than Tconvs. Moreover, we have found that CD69^+^ Tregs are more suppressive than their CD69^−^ counterparts. Collectively, these data indicate superior activation of Tregs in the tumor microenvironment, promoting their suppressive ability and selective proliferation at this site.

## INTRODUCTION

CD4^+^ Foxp3^+^ regulatory T cells (Tregs) serve to maintain immune homeostasis, prevent autoimmunity and limit immunopathology [1]. It is also well known from studies of mouse models and cancer patients, that Tregs are enriched in tumors where they often facilitate disease progression [2–5]. We have used a mouse model of chemical carcinogenesis whereby fibrosarcomas are induced *in vivo* following administration of the chemical carcinogen, 3-methylcholanthrene (MCA) to identify factors promoting enrichment of intra-tumoral Tregs. This model is useful and relevant as there is a highly significant enrichment of Tregs within the fibrosarcomas, and depletion of these Tregs results in T cell mediated control of tumor progression [4, 6, 7].

Interleukin-2 (IL-2), secreted primarily by CD4^+^ Foxp3^−^ T cells, plays a crucial role in maintaining immune tolerance. Tregs do not produce IL-2 but constitutively express CD25 (part of the high affinity IL-2 receptor), suggesting they may have the ability to compete for the IL-2 resource provided by other T cells. Indeed, under homeostatic conditions, the frequency of Tregs in the periphery is regulated by the number of IL-2 producing T cells [8, 9]. The tightly controlled ratio of Tconv to Treg under normal conditions is significantly perturbed in the microenvironment of tumors in mice and humans, where Tregs can represent up to 40-50% of the CD4^+^ T cell pool [4, 5]. It is possible therefore that IL-2 is limited in the tumor microenvironment and that the perturbation in Tconv to Treg ratios reflects competition for limited IL-2 at this site. There is also a growing consensus that Tregs differentiate into distinct lineages optimised in their ability to suppress specific Tconv subsets [10–13]. Thus, the demonstration that T-bet^+^ CXCR3-expressing Tregs accumulate in human ovarian cancer has lent support to the theory that Tregs must ‘mirror’ the T_H_1-orientated anti-tumor response in order to effect immunosuppression [14].

In the study described herein we investigate whether expression of T-bet and / or competition for IL-2 is important for Treg-mediated suppression of tumor immunity. We also explore potential drivers of Treg accumulation in tumors, identifying superior activation and proliferation of intra-tumoral Tregs as key factors underpinning their ability to dominate this highly immunosuppressed site.

## RESULTS

### Intra-tumoral Foxp3^+^ Tregs proliferate more than Foxp3^−^ Tconvs

**Figure 1:**
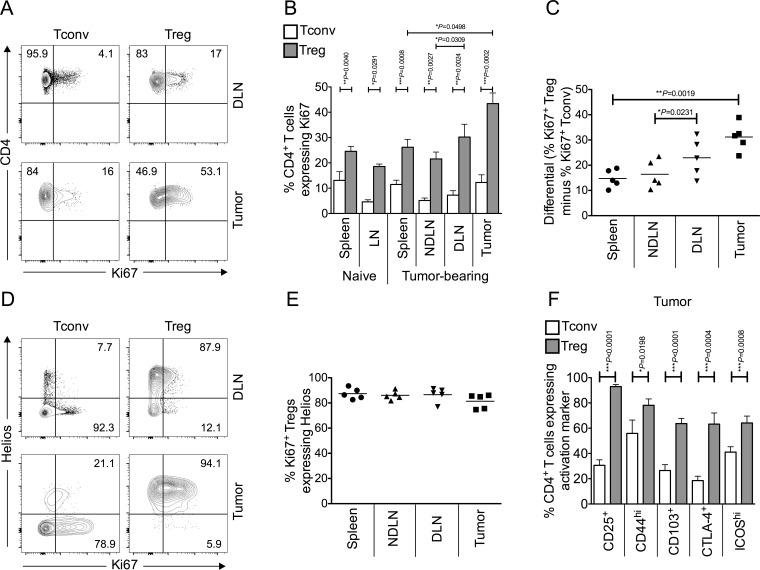
Tumor infiltrating Foxp3^+^ CD4^+^ Tregs are more proliferative and activated than conventional Foxp3^−^ CD4^+^ T cells, and are thymus-derived Helios^+^ Tregs Single cell suspensions prepared from spleen, lymph nodes and tumor of naïve or tumor-bearing mice were stained using CD4-, Foxp3-, Ki67- and Helios-specific monoclonal antibodies and analysed by flow cytometry. **1A.** Representative flow cytometry plots from one animal showing proportion of Ki67^+^ proliferating Tconvs and Tregs, once gated on CD4^+^ Foxp3^−^ cells (left panels) or CD4^+^ Foxp3^+^ cells (right panels), in tumor draining lymph node (DLN), and tumor. **1B.** Proportion of CD4^+^ Foxp3^−^ Tconvs (white bars) and CD4^+^ Foxp3^+^ Tregs (grey bars) expressing Ki67 in spleen, lymph node (LN), tumor non draining lymph node (NDLN), DLN, and tumor. *N* = 6 naïve; *N* = 5 tumor-bearing mice. Data are represented as mean ± SEM. Statistical significance was determined by individual paired *t*-tests between Tconvs and Tregs in each compartment, and Tregs in tumor and spleen, and NDLN and DLN, of tumor-bearing animals. The most highly significant difference was found between Tconvs and Tregs within tumors. **1C.** A dot plot showing the differential (% of Ki67^+^ Foxp3^+^ Tregs minus % of Ki67^+^ Foxp3^−^ Tconv) Ki67 expression in spleen, NDLN, DLN and tumor. *N* = 5. Data are represented as individual data points plus mean. Statistical significance was determined by individual paired *t*-tests. **1D.** Representative flow cytometry plots from one animal showing proportion of Ki67^+^ proliferating Tconvs and Tregs expressing Helios, gated on CD4^+^ Foxp3^−^ cells (left panels) or CD4^+^ Foxp3^+^ cells (right panels), in DLN, and tumor. Numbers represent the proportion of either Ki67^+^ Tconv (left panels) or Ki67^+^ Treg (right panels) that also express the marker Helios, gated on CD4^+^ Foxp3^−^ Ki67^+^ cells (left panels) or CD4^+^ Foxp3^+^ Ki67^+^ cells (right panels). **1E.** A dot plot showing that the vast majority of CD4^+^ Foxp3^+^ Ki67^+^ Tregs also express Helios in spleen, LN, NDLN, tumor draining lymph node (DLN), and tumor. *N* = 5. Data are represented as individual data points plus mean. Statistical significance was determined by individual paired *t*-tests. **1F.** Proportion of CD4^+^ Foxp3^−^ Tconvs (white bars) and CD4^+^ Foxp3^+^ Tregs (grey bars) expressing various activation markers in the tumor. *N* = 10 for CD25, 9 for CD44, 13 for CD103, 8 for CTLA-4, 12 for ICOS. Data are represented as mean ± SEM. Statistical significance was determined by individual paired *t*-tests.

Tregs are highly enriched within the microenvironment of MCA-induced tumors, reaching 40-50% of the CD4^+^ T cell pool [4, 7, 15]. To determine why this should be the case, we initially measured the proliferative status of Tconv and Treg within tumor-bearing mice by Ki67 expression. Ki67 is a nuclear protein expressed during all active phases of the cell cycle and hence is used as a marker of cellular proliferation [16]. A higher proportion of Tregs expressed Ki67 compared to Tconv cells within all of the sites (spleen, lymph nodes and tumor), in both tumor-bearing and naïve mice (Figure 1A–1B). Notably, by far the most significant difference between the proportions of proliferating Tconv and Treg cells was observed within the tumor (Figure 1C).

### Highly proliferative tumor-infiltrating Foxp3^+^ Tregs are thymus-derived Helios^+^ T cells

TCR clonotypes are largely distinct between Foxp3^−^ and Foxp3^+^ CD4^+^ tumor-infiltrating T cell sub-populations, indicating that conversion does not account for intra-tumoral Treg-enrichment and supporting the hypothesis that Tregs within the tumor are thymus-derived Tregs (tTregs) [17]. The Ikaros transcription factor family member, Helios, is expressed predominantly in tTregs [18]. As shown in Figure 1D and 1E, Helios staining was observed mainly in the Foxp3^+^ population, and indeed, the vast majority of proliferating Foxp3^+^ cells also express Helios. These data confirm that the highly proliferative Tregs in the tumor are thymus-derived Helios expressing Tregs.

In order to assess the activation status of intra-tumoral Tregs, flow cytometric analysis of the expression of a number of markers associated with T cell activation were examined on both intra-tumoral Tregs and Tconvs (CD25, CD44, CD103, CTLA4, ICOS). Tregs within the tumor demonstrated significantly higher expression levels of all activation markers studied than Tconvs (Figure 1F). These data confirm that Foxp3^+^ tTregs within the tumor are not only significantly more proliferative but also exhibit a more activated phenotype than Tconvs at this site.

### Tumor-infiltrating Tconvs and Tregs are enriched for inflammation-seeking “T_H_1-like” cells

**Figure 2:**
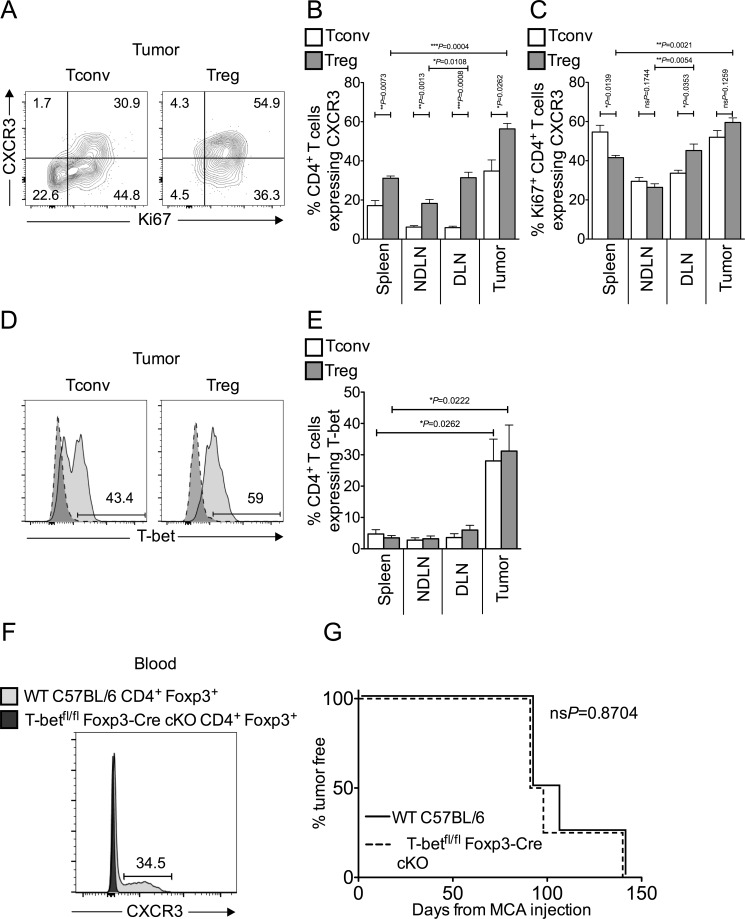
Tumor-infiltrating Tconvs and Tregs are enriched for inflammation-seeking “T_H_1-like” cells Single cell suspensions prepared from spleen, NDLN, DLN and tumor were stained using CD4-, Foxp3-, Ki67-, CXCR3- and T-bet-specific monoclonal antibodies and analysed by flow cytometry. **2A.** Representative flow cytometry plots from one animal showing proportion of Ki67^+^ proliferating Tconvs and Tregs that also express the chemokine receptor CXCR3, once gated on CD4^+^ Foxp3^−^ cells (left panels) or CD4^+^ Foxp3^+^ cells (right panels), in tumor. **2B.-2C.** Proportion of CD4^+^ Foxp3^−^ Tconvs (white bars) and CD4^+^ Foxp3^+^ Tregs (grey bars) (B), and those expressing Ki67 (C), that also express CXCR3 in spleen, NDLN, DLN, and tumor. *N* = 5. Data are represented as mean ± SEM. Statistical significance was determined by individual paired *t*-tests. **2D.** Representative flow cytometry plots from one animal showing proportion of T cells that express the transcription factor T-bet, once gated on CD4^+^ Foxp3^−^ Tconvs (left panels) or CD4^+^ Foxp3^+^ Tregs (right panels), in tumor (pale grey peak, solid outline), relative to the isotype matched control sample (dark grey peak, dashed outline). **2E.** Proportion of CD4^+^ Foxp3^−^ Tconvs (white bars) and CD4^+^ Foxp3^+^ Tregs (grey bars) expressing T-bet in spleen, NDLN, DLN, and tumor. *N* = 5. Data are represented as mean ± SEM. Statistical significance was determined by individual paired *t*-tests and Tregs in tumor and spleen. **2F.** A representative flow cytometry plot from blood samples of one Wild Type C57BL/6 mouse (dark grey peak, solid outline) and one T-bet^fl/fl^Foxp3-Cre conditional KO mouse (pale grey peak, solid outline), showing the proportion of Foxp3^+^ Tregs that express CXCR3. Cells are gated on live CD4^+^ Foxp3^+^ lymphocytes. The number represents the proportion of cells that express CXCR3 in Wild Type C57BL/6 blood. **2G.** Incidence of MCA-induced tumors, as measured by percent of animals tumor free X days after MCA injection. Solid line represents Wild Type C57BL/6 mice; dashed line represents T-bet^fl/fl^Foxp3-Cre conditional KO mice. *N* = 4. Statistical significance was determined by a log rank (Mantel-Cox) test.

T_H_1 and cytotoxic T lymphocyte (CTL) responses limit progression of MCA-induced fibrosarcomas after Treg depletion [4, 7, 19, 20]. Previous studies have reported that Tregs can express the chemokine receptor CXCR3 in response to T_H_1 polarising cues, and that these CXCR3^+^ Tregs accumulate at peripheral sites of inflammation to specifically suppress T_H_1 and CTL responses [10, 11, 14, 21]. When we analysed tumor-infiltrating T cells for CXCR3 expression, a high proportion (approximately 60%) of tumor-infiltrating Tregs were CXCR3^+^ (Figure 2A–2B). Furthermore, the majority of Ki67^+^ proliferating Tregs in the tumor co-expressed CXCR3, as was true for intra-tumoral Tconvs (Figure 2A–2C). We also found T-bet expression in a proportion of both intra-tumoral Tconv and Treg cells (Figure 2D–2E).

It was hypothesised that if, as suggested in the literature [10, 11], T-bet expression is necessary for the ability of Tregs to suppress T_H_1 responses and promote tumor growth, then tumor development should be better controlled in the absence of T-bet expressing Tregs. We used mice genetically engineered to lack T-bet expression in Foxp3^+^ cells (T-bet^fl/fl^Foxp3-Cre conditional KO mice) [22]. As CXCR3 expression is under the control of T-bet in Treg cells, Treg in T-bet^fl/fl^Foxp3-Cre conditional KO mice also display abolished CXCR3 expression [11, 22]. We confirmed the lack of CXCR3 expression in Foxp3^+^ Tregs at the protein level by flow cytometric analysis of blood (Figure 2F). To test the hypothesis, we compared the incidence of MCA-induced tumors in T-bet^fl/fl^Foxp3-Cre conditional KO and WT C57BL/6 mice and found tumor incidence was comparable between the groups (Figure 2G). Thus, whilst these data show that in MCA-induced tumors, approximately 50% of tTregs are “T_H_1-like”, we have no evidence that T-bet is required for Treg-mediated suppression in the tumor environment. Moreover, proportions of intra-tumoral Tregs were comparable in T-bet^fl/fl^Foxp3-Cre conditional KO and WT mice (data not shown).

### Competition for IL-2 does not account for the enrichment of intra-tumoral Foxp3^+^ Tregs

**Figure 3:**
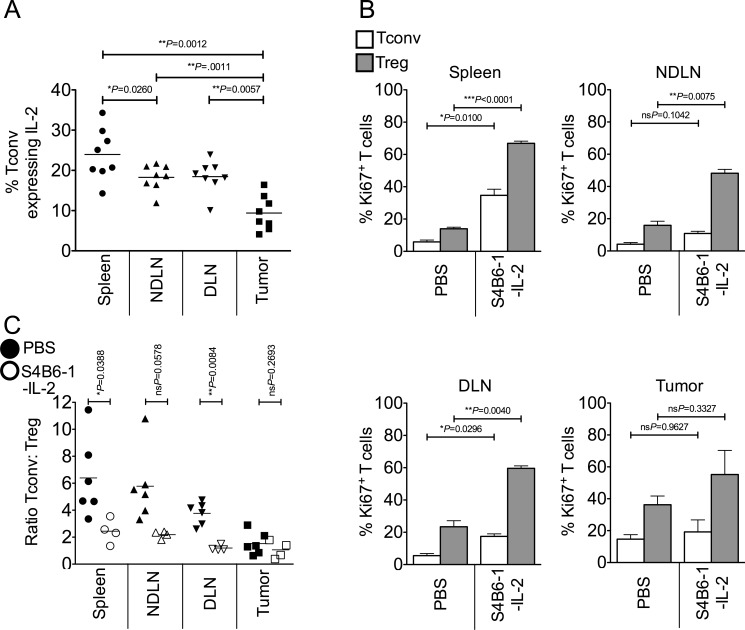
Competition for IL-2 does not account for the enrichment of intra-tumoral Foxp3^+^ Tregs Single cell suspensions prepared from spleen, NDLN, DLN and tumor from PBS-treated or anti-IL-2/IL-2 complex (S4B6-1) treated animals, were stained using CD4-, Foxp3-, CD8-, Ki67- and IL-2-specific monoclonal antibodies and analysed by flow cytometry. **3A.** A dot plot showing the proportion of CD4^+^ Foxp3^−^ Tconvs expressing intra-cellular IL-2 in spleen, NDLN, DLN, and tumor of non-treated animals. *N* = 8. Data are represented as individual data points plus mean. Statistical significance was determined by individual paired *t*-tests. **3B.** Proportion of CD4^+^ Foxp3^−^ Tconvs (white bars) and CD4^+^ Foxp3^+^ Tregs (grey bars) expressing Ki67 in spleen, NDLN, DLN, and tumor, of PBS-treated or anti-IL-2/IL-2 complex (S4B6-1) treated animals. Data are represented as mean ± SEM. Statistical significance was determined by individual paired *t*-tests. **3C.** Ratio of CD4^+^ Foxp3^−^ Tconvs to CD4^+^ Foxp3^+^ Tregs in spleen, NDLN, DLN, and tumor, in animals treated with anti-IL-2/IL-2 complexes (S4B6-1; open shapes) or control animals treated with phosphate buffered saline (PBS; closed shapes). Data are represented as individual data points plus mean. Statistical significance was determined by individual paired *t*-tests.

We postulated that competition for IL-2 represents a key component of intra-tumoral Treg proliferation and subsequent enrichment. In line with this hypothesis, we found that a lower proportion of intra-tumoral Tconv expressed intra-cellular IL-2 compared to secondary lymphoid organs (Figure 3A). To determine whether provision of IL-2 would restore homeostatic ratios of Tregs and Tconvs in the tumor, we injected tumor-bearing mice with anti-IL-2/IL-2 complexes (S4B6-1-IL-2 which binds CD122, stimulating both Tregs and Tconvs) [23] which resulted in a significant increase in proliferation of Tregs and Tconvs in lymphoid tissues but not the tumor (Figure 3B). Accessibility of the S4B6-1-IL-2 complex to the tumor mass was not an issue as the proportion of Ki67^+^ proliferating CD8^+^ T cells in the tumor significantly increased following complex administration (mean of 26.8% for non-treated versus mean of 74.6% for S4B6-1-IL-2 treated; ***P = 0.0010 by paired *t* test; data not shown). Furthermore, whereas administration of this anti-IL-2/IL-2 complex altered the ratio of Tconvs to Tregs in favour of Tregs in secondary lymphoid tissues, the ratio of Tconvs to Tregs in the tumor did not change (Figure 3C). These data indicate that IL-2 is not a limiting factor for proliferation of Tregs or Tconvs in the tumor and that increasing the availability of IL-2 has no impact on the ratio of Tregs to Tconvs at this site.

### CD69 is highly expressed on intra-tumoral Foxp3^+^ Tregs and denotes Tregs with superior suppressive capacity

The data presented thus far indicate that the more activated phenotype of intra-tumoral Tregs is linked to superior proliferation and hence their enrichment within the tumor-infiltrating T cell pool. To further address the implications of superior Treg activation within the tumor microenvironment we turned our attention to the early activation marker, CD69 [24] as it has recently become apparent that CD69, through reciprocal regulation of sphingosine 1-phosphate receptor-1 (S1P1), retains CD69^+^ T cells at the site of antigen [25–27]. Moreover, CD69^+^ Foxp3^+^ Tregs are reportedly more efficient at limiting inflammation than their CD69^−^ counterparts [28], implying that CD69 acts as a negative regulator of T cell activation.

**Figure 4:**
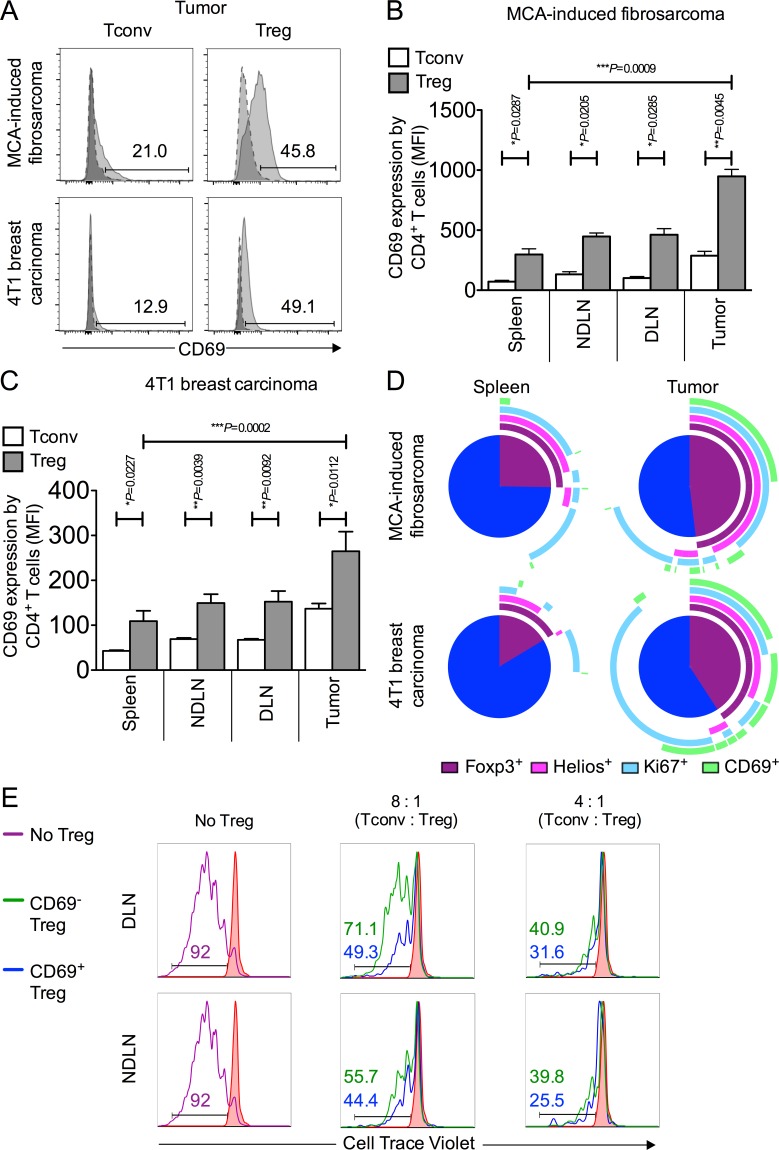
CD69 is highly expressed on intra-tumoral Foxp3^+^ Tregs and denotes superior suppressive capacity **4A.-4D.** Single cell suspensions prepared from spleen, NDLN, DLN and tumor were stained using CD4-, Foxp3-, and CD69-specific monoclonal antibodies and analysed by flow cytometry. **4A.** Representative flow cytometry plots from one animal per model showing proportion of CD4^+^ Foxp3^−^ Tconvs and CD4^+^ Foxp3^+^ Tregs that express CD69, once gated on CD4^+^ Foxp3^−^ (left panels) or CD4^+^ Foxp3^+^ (right panels), in tumor (pale grey peak, solid outline), relative to the isotype matched control sample (dark grey peak, dashed outline). A tumor of the MCA-induced fibrosarcoma model is shown in the top panel; a tumor of the 4T1 breast carcinoma model is shown in the bottom panel. **4B.-C.** Proportion of CD4^+^ Foxp3^−^ Tconvs (white bars) and CD4^+^ Foxp3^+^ Tregs (grey bars) expressing CD69 in spleen, NDLN, DLN, and tumor, expressed as Mean Fluorescence Intensity (MFI), for the MCA-induced fibrosarcoma model (B) and the 4T1 breast carcinoma model (C). *N* = 3 (B), *N* = 8 (C). Data are represented as mean ± SEM. Statistical significance was determined by individual paired *t*-tests. **4D.** Representative pie charts depicting relative co-expression of Foxp3, Helios, Ki67 and CD69 by CD4^+^ T cells in spleen and tumor samples from one animal of the MCA-induced fibrosarcoma model (top panel) and one animal of the 4T1 breast carcinoma model (bottom panel). Each arc represents one of the four individual markers, as shown in the key included in the figure. **4E.** Sorted Tconv and Treg populations were co-cultured in an *in vitro* Treg suppressor assay after which populations were analysed for proliferation by flow cytometry. Histograms showing the proportion of divided Tconv cells according to dilution of CellTrace Violet dye (x axis) with no CD3/CD28 stimulation (filled in red peak), with stimulation (purple peak), with stimulation in the presence of CD69^−^ Tregs (green peak), and with stimulation in the presence of CD69^+^ Tregs (blue peak). Ratios of Tconv : Treg are shown above each set of histograms. Numbers on histograms represent % divided cells, colour-coded for each condition. Data are shown for CD4^+^ CD25^hi^ CD127^lo^ CD69^+^ or CD69^−^ Tregs isolated from (DLN, top) and (NDLN, bottom).

We compared CD69 expression on MCA-tumor-derived Foxp3^−^ and Foxp3^+^ CD4^+^ T cells and found a striking difference in that the proportion of Foxp3^+^ CD4^+^ T cells (mean of 35.4% ± 7.0 SEM) was at least double that of Foxp3^−^ CD4^+^ cells (mean of 13.1% ± 3.0 SEM) (Figure 4A). The same pattern was observed when we compared CD69 expression on Tregs and Tconvs isolated from tumors of the 4T1 breast carcinoma model. These data corroborated our findings in the MCA tumor model, as again a significantly higher proportion of tumor derived Foxp3^+^ Tregs expressed CD69 (mean of 37.1% ± 9.3 SEM) relative to tumor derived Foxp3^−^ Tconv (mean of 13.9% ± 2.3 SEM) (Figure 4A). These highly significant differences in CD69 expression between Tregs and Tconvs within both types of tumor is most striking when analysed by Mean Fluorescence Intensity (MFI) measurement (Figure 4B–4C). The significant enrichment of CD69-expressing Tregs in both MCA and 4T1 tumor models is presented using pie charts displaying relative co-expression of markers Helios, Ki67 and CD69 in Figure 4D.

We next sought to compare the suppressive capacity of CD69^+^ and CD69^−^ Tregs. CD4^+^ CD25^lo^ Tconvs were sorted from lymphoid tissue and co-cultured in the presence of anti-CD3 and anti-CD28 beads with either CD69^+^ or CD69^−^ Tregs sorted from tumor draining or non tumor draining lymph nodes, at varying Tconv : Treg ratios. Treg were sorted as CD4^+^ CD25^hi^ CD127^lo^ (CD69^+^ or CD69^−^) cells. After 120 hours of culture, proliferation of labelled Tconvs was measured by flow cytometry (Figure 4E). Strikingly, CD69^+^ Tregs, recovered from tumor draining and non-draining lymph nodes, were more suppressive of Tconv proliferation than their CD69^−^ counterparts. We observed that CD69^+^ Tregs are 1.3 x and 2 x more suppressive than CD69^−^ Tregs at a Tconv : Treg ratio of 8 : 1 in the NDLN and DLN, respectively. Similarly, CD69^+^ Tregs are 1.3 x and 1.2 x more suppressive than CD69^−^ Tregs at a Tconv : Treg ratio of 4 : 1 in the NDLN and DLN, respectively (Figure 4E). These data are indicative of a superior ability of CD69^+^ Foxp3^+^ Tregs to suppress proliferation of anti-tumor Tconv cells, in relation to CD69^−^ Foxp3^+^ Tregs.

## DISCUSSION

The observation that Tregs are enriched in tumors has been made using numerous mouse models and through the study of cancer patients [2–5]. The aim of this study was to investigate the mechanism of this enrichment, employing a carcinogen-induced tumor model to examine Treg phenotype with respect to their intra-tumoral accumulation and suppressive function.

A side-by-side analysis of the proliferation and survival characteristics of intra-tumoral Tregs and Tconvs revealed significant differences between the two T cell populations. The proportion of Ki67^+^ Tregs was significantly higher than Tconvs in spleens and lymph nodes, corroborating previous observations [29]. However, the novel data presented herein indicate that the extent of this difference is most striking in T cells recovered from the tumor mass. Enhanced proliferation was not accompanied by greater cell death within the Treg population (data not shown) thus superior proliferation of Tregs must contribute to their enrichment within the tumor. Preferential proliferation and accumulation of Tregs in the tumors does not reflect increased competition for IL-2, as increasing the availability of IL-2 has no impact on the ratio of Tregs to Tconvs at this site. These findings also indicate that Tconvs are not held in check through competition for IL-2. Whilst increasing the availability of IL-2 had no impact on proliferation of intra-tumoral Tregs or Tconvs, a significant increase in proliferation of CD8^+^ T cells was observed. Whilst the reason for this disparity is unclear, it is possible that intratumoral CD4^+^ T cells (unlike CD8^+^ T cells at the same site) are highly differentiated, and hence no longer proliferate in response to IL-2.

The proliferating Tregs in tumors expressed both CXCR3 and T-bet, in keeping with a “T_H_1-like” lineage, and Helios, a marker thought to define thymus-derived Tregs. We have no evidence however that T-bet expression defines Tregs with a superior capacity to suppress anti-tumor immune responses, which in the case of the MCA-induced fibrosarcomas described herein is a T_H_1-orientated response [6, 19, 20]. These data are in contrast to the previous observations made by Koch and colleagues, where T-bet deficient Tregs were inferior to wild-type Tregs during control of T_H_1-mediated multi-organ autoimmunity [11]. However, several studies have reported either no alteration in suppressive capacity of Tregs when T-bet is absent [30, 31] or even superior suppressive function of T-bet deficient Tregs [32]. A recent study using experimental autoimmune encephalomyelitis (EAE) reported that lack of T-bet expression in Foxp3^+^ Tregs did not hinder their infiltration of the inflamed central nervous system, which is important for EAE resolution. T-bet deficient Tregs were also capable of preventing the development of T cell-driven colitis [22]. These data together with our own tumor-related data question the relevance of peripheral differentiation of Tregs as a means of steering their function towards selective inhibition of a particular type of immune response. It is possible that expression of T-bet, and indeed other master transcription factors, by Tregs could simply be a reflection of chronic exposure to pro-inflammatory, activating signals in certain disease settings, rather than a requirement for specific functionality.

This study also examined a role for CD69, a C-type lectin, upregulated on T cells upon antigen stimulation [24]. Paradoxically, CD69^−/−^ mice exhibit enhanced tumor immunity and enhanced susceptibility to autoimmune disease raising the possibility that the molecule is a negative regulator of T cell activation [33, 34]. It is possible that this effect of CD69 is mediated by Foxp3^+^ Tregs, as CD69^+^ Foxp3^+^ Tregs have been shown to limit inflammation more efficiently than CD69^−^ Tregs [28]. This effect of CD69 may relate to reciprocal regulation of CD69 and S1P1 signalling [25–27]. As S1P1 signalling upregulates the Akt-mTOR pathway, resulting in diminished suppressor activity in Tregs [35], it follows that CD69^+^ Treg would exhibit increased suppressor activity. Our findings support this premise. It has also become apparent that CD69 plays a role in retaining T cells at the site of antigen, through preventing expression of S1P1, thereby stopping lymphocyte egress from the site of antigen recognition [25–27]. Thus, in the case of the tumors described herein, the consequence of CD69 expression on Tregs may be the retention of tumor-infiltrating super-suppressors thereby ensuring Treg dominance within the tumor microenvironment.

In conclusion, we have eliminated competition for a limited IL-2 resource as a means by which Tregs become significantly enriched in tumors. Despite the discovery of an accumulation of T_H_1-like Tregs in tumors, this feature did not contribute to the functional ability of Tregs to control anti-tumor immune responses. Instead, our observations highlight increased proliferation of highly suppressive CD69^+^ Tregs as a mechanism by which Tregs exert dominance within the tumor microenvironment.

## MATERIALS AND METHODS

### Mice

Mice expressing the primate diphtheria toxin receptor (DTR) under the control of the Foxp3 promoter (Foxp3^DTR^ mice) were obtained from Professor Alexander Rudensky and have been described previously [36]. Foxp3^DTR^ mice were backcrossed with C57BL/6 mice for 10 generations and housed in accordance with UK Home Office regulations under specific pathogen free conditions. In this study, mice were not administered diphtheria toxin so had normal proportions of Foxp3^+^ lymphocytes. T-bet^fl/fl^Foxp3-Cre conditional KO mice were gratefully received from Professor Stephen Anderton and have been described previously [22].

### Tumor induction

For tumor induction, Foxp3^DTR^ mice or T-bet^fl/fl^Foxp3-Cre conditional KO mice, aged 8 to 15 weeks, were injected subcutaneously in the left hind leg with 400μg of 3-methylcholanthrene (MCA; Sigma-Aldrich) in 100μl of olive oil under general anaesthetic, as previously described [17]. Mice were monitored for tumor development weekly for up to 2 months and daily thereafter. Tumor-bearing mice were culled when their tumors were between 1cm and 2cm in diameter, typically 100-150 days after MCA injection.

### Administration of Anti-IL-2 / IL-2 complexes

For treatment with Anti-IL-2/IL-2 complexes, 5μg rIL-2 (PeproTech) was incubated with 50μg of anti-IL-2 mAb (S4B6-1; Bio-Xcell) for 10 minutes at room temperature, prior to injection. Following development of a palpable tumor, complexes were administered by intraperitoneal injection in 200μl of solution every day for three consecutive days before sacrifice.

### Preparation of single cell suspensions for antibody staining and flow cytometry

Single cell suspensions of spleen, and inguinal lymph nodes (LN) were prepared by homogenising tissues in multiwell plates using the back of a syringe plunger. The inguinal LN from the tumor (left) side of the mouse was taken as the tumor draining LN and the contralateral inguinal LN was considered to be a non-draining LN. Dissected tumors were initially diced into a pulp using a scalpel before homogenisation. Homogenised tissues were resuspended in complete RPMI (RPMI [Invitrogen] supplemented with 2mM L-glutamine, 1mM sodium pyruvate, 50μg/ml penicillin streptomycin and 10% foetal calf serum) and filtered through 70μM cell strainers (BD Biosciences). Single cell suspensions were centrifuged at approximately 450g for 5 minutes and cell pellets were resuspended in FACS buffer (phosphate buffered saline (PBS) containing 5mM EDTA and 2% foetal calf serum). Prior to staining, spleen and tumor cell pellets were subjected to red blood cell lysis by resuspending cell pellets in 5ml RBC Lysis buffer (1/10 dilution in dH_2_O; BioLegend) for 90 seconds at room temperature.

### Cell surface staining

Single cell suspensions were plated in 96 round-bottomed well plates at 0.5-1 million cells per well. Cells were washed twice in PBS prior to live/dead staining by adding 3μl (or 6μl for tumor cell pellets) of LIVE/DEAD Aqua (diluted 1:10 in PBS; Invitrogen) for 15 minutes in the dark at room temperature. Cells were washed twice in FACS buffer prior to Fc Receptor blocking by adding 5mg/ml anti-CD16/32 (93; eBioscience) antibody to the cells and incubating for 10 minutes at 4°C in the dark. After two washes in FACS buffer, cells were stained with 25-50μl of antibodies diluted in FACS buffer and incubated at 4°C in the dark for 10-15 minutes. Cell surface antibodies consisted of anti-CD3-PECy5 (17A2; BD Biosciences), anti-CD4-APC-eFluor 780 (RM4-5; eBioscience), anti-CD8-Pacific Blue (53-6.7; BD Biosciences), anti-CXCR3-APC (CXCR3-173; eBioscience) and anti-CD69-FITC (H1.2F3; BD Biosciences).

### Intracellular antigen and cytokine staining

For intracellular cytokine analysis, single cell suspensions (0.5-1 million cells per well) were stimulated in 24 well plates with complete RPMI containing 20 nM phorbol myristate acetate (PMA; Sigma-Aldrich) and 1μg/ml ionomycin (Sigma-Aldrich), at 37°C for 4 hours. After 1 hour of incubation, 1μl/ml of GolgiStop (containing monensin; BD Biosciences) was added to each well. Surface stained cells were stained for intracellular antigen following Fixation/Permeabilisation (Foxp3-staining kit, eBiosciences). Intracellular antibodies consisted of anti-Ki67-FITC (B56; BD Bioscience) or anti-Ki67-BrilliantViolet 421 (16A8; BioLegend), anti-IL-2-PE (JES6-5H4; eBioscience), anti-Foxp3-PE-Cy7 (FJK-16s; eBioscience), and anti-T-bet-PerCPCy5.5 (eBio4B10; eBioscience). For flow cytometric analysis, samples were acquired on a FACS Canto II flow cytometer (BD Biosciences), and were subsequently analysed using FlowJo software.

### *In vitro* Treg suppression assay

Single cell suspensions were stained as detailed above, using Live/Dead Aqua, anti-CD3-PECy5 (17A2; BD Biosciences), anti-CD4-APC-eFluor 780 (RM4-5; eBioscience), anti-CD25-PE (PC61; BD Biosciences), and anti-CD127-BrilliantViolet605 (A7R34; BioLegend) monoclonal antibodies. CD3^+^ CD4^+^ CD25^lo^ Tconvs were sorted from spleen, and CD3^+^ CD4^+^ CD25^hi^ CD127^lo^ CD69^+^ Tregs and CD3^+^ CD4^+^ CD25^hi^ CD127^lo^ CD69^−^ Tregs were sorted from either non-tumor draining lymph nodes or tumor-draining lymph nodes using a customized 20-parameter FACSAria II flow cytometer (BD). Too few Tregs were isolated from pooled tumor samples to perform reliable assays. Post-sort purity checks on practice cell sorts revealed greater than 98% purity.

Sorted Tconvs were stained with CellTrace Violet (Life Technologies) according to the manufacturers' specifications, pelleted and resuspended in warm complete RPMI at a concentration of 2.8 × 10^5^ cells per ml, so as to deliver 14, 000 effector cells in 50μl.

Sorted Tregs were washed and resuspended in warm complete RPMI culture medium (complete RPMI supplemented with β-Mercaptoethanol, MEM NEAA (100X; gibco) and 5mM HEPES buffer solution (gibco)). 14, 000 Tconvs per well were incubated with anti-mouse CD3/CD28 dynabeads (Invitrogen) at a ratio of 1:2 (beads:Tconvs), with titrating numbers of Tregs, in complete RPMI culture medium for 120 hours. After 72 hours, 100μl of fresh, warm complete RPMI culture medium was added. Following culture, cells were stained with Live/Dead Aqua, anti-CD3-PE-Cy5, anti-CD4-APC eFluor 780 and anti-CD25-PE, as detailed above. Dilution of CellTrace Violet was measured on a FACS Canto II flow cytometer (BD Biosciences). CD25^hi^, CellTrace Violet negative Tregs were excluded from analysis.

## Abbreviations

Treg: CD4^+^ Foxp3^+^ Regulatory T cell
Foxp3: Forkhead box 3 transcription factor
MCA: 3-methylcholanthrene
Tconv: CD4^+^ Foxp3^−^ conventional T cell
NDLN: non-tumor draining lymph nodes
TDLN: tumor draining lymph nodes
tTreg: thymus-derived CD4^+^ Foxp3^+^ Regulatory T cell
T_H_1: T helper cell type 1
IL-2: Interleukin 2
